# Monitoring of RSV-A and RSV-B Circulation in Poland Across Three Post-Pandemic Seasons (2022–2025)

**DOI:** 10.3390/v18030321

**Published:** 2026-03-04

**Authors:** Katarzyna Łuniewska, Piotr Rzymski, Barbara Poniedziałek, Karol Szymański, Katarzyna Kondratiuk, Emilia Czajkowska, Bartosz Mańkowski, Lidia B. Brydak

**Affiliations:** 1 Department of Virology, National Institute of Public Health, NIH-National Research Institute, 00-791 Warsaw, Poland; kluniewska@pzh.gov.pl (K.Ł.); kszymanski@pzh.gov.pl (K.S.); ktomczuk@pzh.gov.pl (K.K.); eczajkowska@pzh.gov.pl (E.C.); bmankowski@pzh.gov.pl (B.M.); lbrydak@pzh.gov.pl (L.B.B.); 2 Department of Environmental Medicine, Poznan University of Medical Sciences, 60-806 Poznań, Poland; bpon@ump.edu.pl

**Keywords:** respiratory syncytial virus, respiratory infections, molecular epidemiology, viral coinfections, public health, RSV vaccines

## Abstract

Respiratory syncytial virus (RSV) is a significant cause of respiratory infections across all ages. However, data on the circulation of its antigenic subgroups, RSV-A and RSV-B, remain limited in certain regions, including Poland. Therefore, this study provides the first molecular insight into the post-pandemic circulation of RSV subgroups in Poland. We analyzed 377 RSV-positive respiratory samples collected across Poland during three consecutive seasons (2022/23, 2023/24, and 2024/25) using qRT-PCR to determine subgroup distribution. An equal prevalence of RSV-A and RSV-B was observed in 2022/23, followed by RSV-A predominance in 2023/24 and a shift to RSV-B dominance in 2024/25. Individuals infected with RSV-A were significantly younger than those infected with RSV-B, a pattern evident in the latter two seasons but not in 2022/23. In general, adults (≥18 years) had higher odds of RSV-B infection (OR = 2.35, 95% CI: 1.44–3.84; *p* = 0.006). Coinfections with both subgroups increased from 5% in 2022/23 to approximately 15% in later seasons, and were more frequent in women. Coinfections with influenza viruses or SARS-CoV-2 were infrequent and showed no statistically significant differences between seasons. The findings of the present study highlight dynamic, region-specific RSV epidemiology and underscore the importance of sustained molecular surveillance to inform public health preparedness and guide emerging RSV immunization strategies in Poland.

## 1. Introduction

Respiratory syncytial virus (RSV) is a leading cause of acute lower respiratory tract infections across the lifespan and remains a significant global public health concern. RSV is the most common viral cause of bronchiolitis and pneumonia in infants and young children worldwide, accounting for substantial morbidity, hospitalizations, and mortality, particularly in infants under six months of age [1]. In addition to its well-recognized pediatric burden, RSV is increasingly acknowledged as a significant pathogen in older adults and individuals with chronic comorbidities, in whom it can cause severe respiratory disease, exacerbate underlying conditions, and lead to excess hospitalizations and deaths [2,3]. The global impact of RSV is therefore comparable to that of influenza in vulnerable populations, underscoring the need for continued epidemiological monitoring [4,5].

RSV is an enveloped, negative-sense RNA virus belonging to the Pneumoviridae family and is classified into two major antigenic subgroups, RSV-A and RSV-B, primarily based on variability within the attachment (G) glycoprotein [6]. Although both subgroups co-circulate during seasonal epidemics, their relative prevalence may vary geographically and temporally, with periodic shifts in dominance observed between epidemic seasons [7,8]. Differences in transmissibility, immune-escape potential, and disease severity have been reported between RSV-A and RSV-B, although findings remain inconsistent across studies [8]. Both RSV subgroups are clinically relevant and capable of causing the full spectrum of disease, ranging from mild upper respiratory tract infection to severe bronchiolitis and pneumonia requiring hospitalization. Most studies assessing potential clinical differences between RSV-A and RSV-B have been conducted in pediatric populations and have generally reported no consistent differences in disease severity. Importantly, coinfections with both subgroups have also been reported, but their epidemiological frequency and clinical significance are not well defined [9].

Comprehensive RSV surveillance is crucial for capturing these patterns and informing public health responses. Historically, RSV surveillance has lagged behind influenza surveillance, particularly in routine subgroup-specific reporting. This gap has become increasingly relevant with the recent introduction of novel RSV immunoprophylaxis strategies. These include long-acting monoclonal antibodies for infants, such as nirsevimab and clesrovimab [10,11], as well as newly licensed vaccines for older adults and maternal immunization to protect young infants through passive immunity. The effectiveness and optimal deployment of these interventions depend on an accurate understanding of RSV epidemiology, including seasonal dynamics, subgroup circulation, and potential shifts in dominance that may influence viral evolution or immune pressure. Establishing robust baseline data on RSV subgroup circulation prior to or during early implementation of these interventions is essential for evaluating their population-level impact and for detecting potential changes in subgroup prevalence driven by selective immune pressure.

Despite increased global focus on RSV prevention, data on RSV subgroups remain limited in many regions. The increased utilization of rapid antigen tests in outpatient and inpatient diagnostics has improved the detection of RSV infections, including in adults; however, these tests do not allow distinction between RSV subgroup [12]. While some countries have reported subgroup-specific circulation and seasonal shifts, systematic data are scarce in Central and Eastern Europe. In Poland, surveillance has primarily detected RSV overall [13,14], without distinguishing between RSV-A and RSV-B, resulting in a significant knowledge gap. As a result, assumptions—such as the presumed dominance of RSV-A—have often been based on data from other regions, even though subgroup prevalence can differ markedly between countries and seasons [8]. This lack of subgroup-resolved data in some regions, such as Poland, limits accurate interpretation of national RSV epidemiology and comparison of post-pandemic trends across Europe.

To address this knowledge gap, we conducted a study to assess the presence and distribution of RSV-A and RSV-B infections, as well as RSV-A/RSV-B coinfections, in Poland across three consecutive epidemic seasons (2022/23, 2023/24, and 2024/25), covering the post-COVID-19 transition and subsequent post-pandemic period. Such a research is especially important since the COVID-19 pandemic substantially disrupted the circulation of various respiratory pathogens, including RSV [15,16,17]. These disruptions may have altered population-level immunity and viral competition, with potential consequences for RSV transmission dynamics and subgroup circulation in the post-pandemic period. Our objectives were to characterize subgroup-specific circulation patterns, evaluate seasonal variability and potential shifts in dominance, and provide baseline epidemiological data for Poland. These findings are intended to support future RSV surveillance efforts and inform the implementation and evaluation of emerging RSV immunoprophylaxis strategies.

## 2. Materials and Methods

### 2.1. Sample Collection

The study included respiratory swab samples that tested positive for RSV, collected between 1 October 2022 and 30 September 2025 by the Voivodeship Sanitary and Epidemiological Stations (VSES) across Poland, ensuring comprehensive nationwide coverage. All specimens were obtained as part of the routine national virological surveillance of respiratory infections in Poland. Detailed clinical metadata, such as hospitalization status, care setting, or symptom severity, were not available in the anonymized surveillance dataset and therefore could not be analyzed. The collected specimens were categorized into three consecutive epidemic seasons: 2022/23, 2023/24, and 2024/25. Information on the sex and age of the individuals was available. In addition, data on confirmed infections with influenza A/B viruses and SARS-CoV-2 were provided. To reflect RSV risk, four age groups were distinguished: 0–5 years, 6–17 years, 18–59 years, and ≥60 years. Although the collection was based on routine surveillance rather than systematic sampling, this approach ensured broad temporal and geographic representation of RSV circulation across three consecutive epidemic seasons. None of the included children received monoclonal anti-RSV antibodies (i.e., nirsevimab or palivizumab), and none were born to mothers vaccinated against RSV. Moreover, none of the adult participants had received RSV vaccination.

### 2.2. RNA Extraction and RSV Subgroup Determination

Viral RNA was extracted from all RSV-positive samples collected by the VSES in the 2022/23, 2023/24, and 2024/25 epidemic seasons using the QIAamp Viral RNA Mini Kit (Qiagen GmbH, Hilden, Germany) according to the manufacturer’s instructions. Prior to the analysis, RNA was eluted in 60 µL of the elution buffer provided with the kit. Molecular diagnostics were performed immediately after isolation to minimize RNA degradation over time. RSV typing was carried out using a qRT-PCR assay with the RealStar RSV RT-PCR Kit 3.0 (Altona Diagnostics GmbH, Hamburg, Germany), following the manufacturer’s instructions. This qualitative assay targets subgroup-specific regions of the RSV genome, allowing simultaneous detection and differentiation of RSV-A and RSV-B. Amplification signals are detected in separate fluorescence channels for each subgroup using specific reporter probes (Cy^®^5 for RSV-A and FAM™ for RSV-B), ensuring high analytical specificity without cross-reactivity. An internal control included in each reaction monitored sample integrity and PCR inhibition.

### 2.3. Statistical Analyses

To assess differences in demographic characteristics and infection patterns, statistical analyses were performed using Statistica software v.15.0 (StatSoft, Tulsa, OK, USA). Statistical significance was defined as a *p*-value < 0.05 for all tests. Categorical variables, including sex distribution, RSV subgroup frequencies (RSV-A, RSV-B, and RSV-A/RSV-B coinfections), and rates of viral coinfections, were compared using Pearson’s chi-square (χ^2^) test. Age was treated as a continuous variable and tested for normality using the Shapiro–Wilk test. Since the assumption of a Gaussian distribution was not met, age comparisons between groups were performed using the non-parametric Mann–Whitney U test, while comparison between three groups was performed using Kruskal–Wallis ANOVA with post hoc Dunn’s test. These tests were applied to compare age distributions between epidemic seasons, between RSV-A- and RSV-B-infected individuals, and between mono-infected and coinfected subjects, both overall and within individual seasons.

## 3. Results

Overall, 130, 112, and 135 RSV-positive samples from the 2022/23, 2023/24, and 2024/25 epidemic seasons, respectively, were subjected to subgroup typing. The characteristics of the individuals from whom these samples were collected are summarized in Table 1. Their age was lower in the 2022/23 and 2023/24 seasons due to a higher percentage of individuals aged 0–5 years; however, the sex distribution did not differ between the analyzed periods.

**Figure 1:**
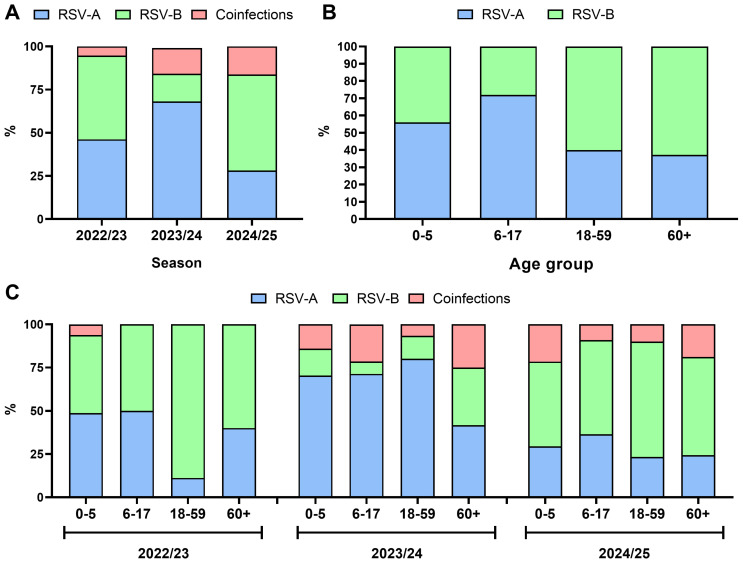
(**A**) Epidemiological shifts in the share of infections with a particular RSV subgroup in Poland (**A**) across three seasons (2022/23, 2023/24, and 2024/25), (**B**) in relation to age across three epidemic seasons jointly, and (**C**) in relation to age group in a particular epidemic season.

The epidemiological shifts in RSV-A and RSV-B across the studied periods are illustrated in Figure 1A. Samples from the 2022/23 season had an equal contribution of RSV-A and RSV-B monoinfections (46.2%, 60/130 and 48.5%, 63/130, respectively; χ^2^ = 0.14, *p* = 0.709), 2023/24 season were dominated by RSV-A monoinfections (68.1%, 77/112; χ^2^ = 63.6, *p* < 0.00001), whereas in 2024/25, a significant shift was observed, with prevalence of RSV-B monoinfections (55.6%, 75/135; χ^2^ = 20.8, *p* < 0.00001). The coinfection rate with RSV-A and RSV-B was the lowest in the 2022/23 season (5.3%, 7/130) and higher but comparable in 2023/24 (15.0%, 17/112) and 2024/25 (16.3%, 22/135, respectively; χ^2^ = 0.06, *p* = 0.811). Coinfections with RSV and SARS-CoV-2 were slightly more frequent in 2023/24 (5.3%, 6/112) than in 2024/25 (1.5%, 2/135) (χ^2^ = 2.9, *p* = 0.09). Coinfections with influenza viruses occurred at a similar frequency across the three seasons (χ^2^ = 1.58, *p* = 0.454), being detected in 3.9% (5/130) of samples in 2022/23—predominantly with influenza type A (80.0%, 4/5)—in 6.2% (7/112) of samples in 2023/24—mainly type A (85.7%, 6/7)—and in 7.4% (10/135) of samples in 2024/25, where type A and type B viruses were equally represented (50% each).

No association was found between sex and infection with a particular RSV subgroup in the studied cohort (*χ*^2^ = 1.64, *p* = 0.200), although coinfections were more frequent in women than in men (16.0% vs. 8.7%, *χ*^2^ = 3.9, *p* = 0.03). Individuals infected with RSV-A were younger than those infected with RSV-B (mean ± SD 14.2 ± 23.15 vs. 23.9 ± 29.6 years, *p* = 0.03), and this difference was also observed when analyzed separately for 2023/24 (15.1 ± 23.3 vs. 23.0 ± 22.5, *p* = 0.02) and 2024/25 (27.1 ± 29.8 vs. 35.9 ± 30.8 years, *p* = 0.015), but not in 2022/23 (5.0 ± 13.7 vs. 10.3 ± 20.4 years, *p* = 0.25). When all three epidemic seasons were analyzed jointly, a tendency was observed in which children and adolescents were more frequently infected with RSV-A, whereas adults, including those aged ≥ 60 years, were more often infected with RSV-B (Figure 1B). In general, adults (≥18 years) had higher odds of RSV-B infection (OR = 2.35, 95% CI: 1.44–3.84; *p* = 0.006). The age-stratified analysis revealed that, although the distribution of RSV subgroups was comparable in the 2022/23 season, RSV-B accounted for a higher proportion of infections among adults aged ≥ 18 years (Figure 1C). Specifically, RSV-B monoinfections among individuals aged 18–59 years and ≥60 years accounted for 88.9% and 60.0%, respectively, in the 2022/23 season; 13.3% and 33.3% in the 2023/24 season; and 66.7% and 56.8% in the 2024/25 season. In addition, although RSV-A generally predominated in the 2023/24 season, individuals aged ≥ 60 years had the highest RSV-B infection rate, at 33.3%, among all age groups. In the subsequent 2024/25 season, infections with RSV-B dominated across all age groups (Figure 1B). The age of those coinfected with RSV-A and RSV-B did not differ significantly from that of monoinfected subjects (21.4 ± 30.3 vs. 18.8 ± 30.3, *p* = 0.803).

## 4. Discussion

This is the first study to report on the distribution of RSV subgroups and seasonal shifts in Poland. It thus fills a critical knowledge gap in the molecular epidemiology of RSV in Central Europe, a region where detailed subgroup-specific surveillance has been limited. Previous studies conducted in Poland focused primarily on the age-stratified seroprevalence of RSV antibodies or RSV-related hospitalizations, primarily in pediatric populations [14,18,19]. In turn, national surveillance data reports RSV infections without subgroup differentiation [13]. At the same time, the increased use of rapid antigen tests in clinical settings further limits our understanding of the molecular landscape in Poland [20]. Therefore, by documenting subgroup dynamics over three consecutive epidemic seasons, this research provides an essential baseline for future longitudinal and genomic analyses.

**Figure 2:**
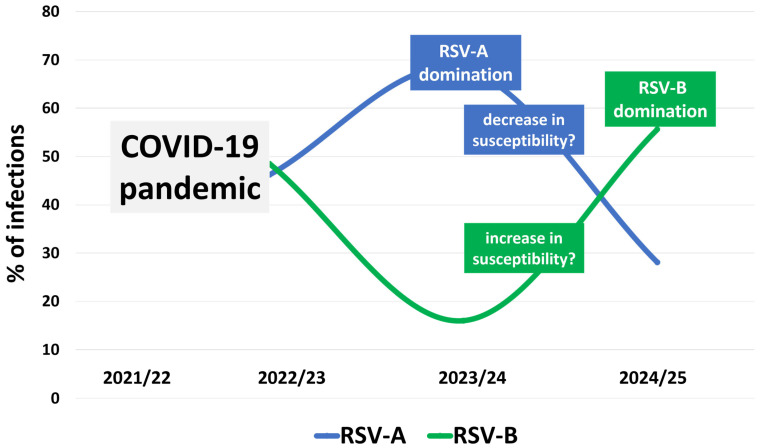
**Schematic representation of epidemiological shifts in the RSV subgroup domination in Poland in 2022–2025.** Following the COVID-19 pandemic, a period during which genomic surveillance of RSV was largely suspended, the 2022/23 epidemic season (the first one after the lifting of all pandemic-related sanitary restrictions) was characterized by a similar circulation of RSV-A and RSV-B. In contrast, the subsequent 2023/24 season showed clear dominance of RSV-A, which may have contributed to increased subgroup-specific population immunity. This was followed in the 2024/25 season by a reduced number of RSV-A infections and a concurrent increase in RSV-B cases, likely reflecting decreased susceptibility to RSV-A and increased susceptibility to RSV-B.

Our research documents post-COVID-19 shifts in the dominance of RSV-A and RSV-B subgroups in Poland. While RSV and influenza differ substantially in their epidemiology, comparable patterns of strain competition and immunity-driven replacement have been described for some influenza viruses, where only one or two strains circulate annually, and previously epidemic viruses do not reappear, likely due to population-level immunity [21]. In our work, during the 2022/23 epidemic season, an equal distribution of viral subgroups was observed in our sample set, which contrasts with observations from other European countries. For example, in Portugal, Germany, and Denmark, the RSV surge was driven predominantly by RSV-B [22,23,24]. In the 2023/24 season, RSV-A was more frequently detected, whereas the 2024/25 season showed a marked increase in RSV-B prevalence (Figure 2). This dynamic alternation in subgroup dominance is a well-known phenomenon in RSV epidemiology [7,8,25]. Still, the timing and scale of the shift in Poland appear distinct in the post-pandemic context. Notably, such an inversion of dominance between consecutive seasons has not always been observed in other European countries (e.g., Croatia) [26], suggesting that the disruption of viral circulation during the COVID-19 pandemic may have temporarily altered subgroup-specific immunity, enabling rapid rebound and replacement once RSV activity resumed [27,28]. Interestingly, this pattern contrasts with reports from other regions, where RSV-B dominated in 2023/24 and a reversal toward RSV-A was seen in 2024/25 (e.g., in several European countries and the United States) [29,30,31]. These discrepancies highlight the highly regional nature of RSV epidemiology, likely influenced by local exposure history, immunity gaps in different age groups, and the random reintroduction of specific viral lineages. Other factors, such as climate, timing of school reopening, and contact patterns, may also affect transmission dynamics, though evidence is inconclusive [7,32,33,34]. Thus, while global surveillance is valuable for comparison, our findings emphasize that subgroup dominance patterns cannot be readily extrapolated across populations, underscoring the need for ongoing, region-specific genomic monitoring to inform public health responses and guide vaccine strategies.

Moreover, these observations highlight a need to consider infectious disease modeling. Traditional compartmental models, such as the Susceptible-Infected-Recovered framework, are foundational for epidemic forecasting but typically assume a single, homogenous pathogen that confers lifelong immunity [35]. The dynamics documented in our study, where RSV-A and RSV-B alternately dominate and can reinfect the same populations, suggest that such simple models may be insufficient for predicting RSV circulation. Instead, the observed patterns point to the need for more complex modeling frameworks that account for partial and waning immunity, as well as potential cross-protection between antigenically distinct subgroups. Incorporating these elements would allow models to better capture the nuanced, region-specific epidemiological behavior described here.

The observed shifts between RSV-A and RSV-B observed in our work likely occurred within the broader context of post-pandemic reconfiguration of the immune landscape. During the COVID-19 pandemic, reduced RSV exposure resulted in immunity gaps, particularly among young children born during periods of low viral circulation [36,37]. This concept, often described as “immunity debt,” remains the subject of ongoing debate and should be interpreted cautiously, as altered epidemic dynamics may also reflect changes in contact patterns, viral interference, or surveillance practices. Nevertheless, reduced prior exposure could have played a role in shaping atypical epidemic timing and subgroup replacement patterns following the resumption of community transmission. Similar post-pandemic rebounds have been reported across Europe, Asia, and the Americas [38,39,40].

Although our molecular analysis was restricted to subgroup determination, the observed alternation could also be linked to viral evolutionary dynamics. Previous genomic studies have shown that the emergence of new genotypes, such as ON1 in RSV-A and BA in RSV-B, can rapidly alter epidemiological patterns and drive regional replacement events [41,42,43]. Continuous molecular surveillance, integrating full-genome sequencing and antigenic data, will therefore be critical in determining whether the RSV-B resurgence in Poland in the 2024/25 season corresponds to globally circulating variants or represents an independent local lineage expansion. Such data would also help evaluate whether specific genotypic transitions contribute to altered transmissibility, immune escape, or virulence.

Another important finding of the present research was the consistently younger age of individuals infected with RSV-A compared with RSV-B, observed both in the pooled analysis and separately in the 2023/24 and 2024/25 epidemic seasons, but not in 2022/23. Notably, this association persisted despite marked differences in the overall age distribution of samples between the 2023/24 and 2024/25 seasons, suggesting that it reflects a genuine subgroup-specific pattern rather than seasonal sampling effects. This finding suggests a higher prevalence of RSV-A among young children, who represent the most immunologically naïve population, or may reflect differential accumulation of subgroup-specific immunity, with older individuals being relatively more susceptible to RSV-B. The exact reasons behind this phenomenon are unknown, lie beyond the scope of the present research, and remain purely speculative. Nevertheless, such observations justify further age-stratified RSV subgroup genomic surveillance, as it is likely to play a role in assessing the effectiveness of public health measures. In contrast, no age difference was observed between mono-infected and RSV-A/RSV-B coinfected individuals, implying that dual infections are not primarily driven by age-related susceptibility.

Although sex was not associated with infection by a particular RSV subgroup, the higher frequency of coinfections observed in women warrants further investigation. Potential explanations for this phenomenon may include sex-related differences in healthcare-seeking behavior, occupational or caregiving-related exposure, or residual sampling bias, although none of these factors could be directly assessed in the present study.

In addition, the present research shows that RSV-A/RSV-B coinfections were relatively uncommon in the 2022/23 season (~5%), but reached higher, comparable levels in the 2023/24 and 2024/25 seasons (15–16%). This pattern contrasts with some earlier reports from other geographic regions, where RSV-A/RSV-B coinfections are generally considered uncommon and typically account for less than 1–3% of RSV detections [9,44]. However, some mixed RSV-A/RSV-B outbreaks have also been recently reported [45]. The relatively higher coinfection rate observed in our study, as well as its increase from the 2023/24 season onwards, may reflect differences in population susceptibility patterns and warrants further investigation, particularly to determine whether mixed RSV-A/RSV-B infections differ in clinical severity. The increase in RSV-A/RSV-B coinfections observed after the 2022/23 season may reflect intensified post-pandemic co-circulation of both RSV subgroups rather than a long-standing endemic feature of RSV epidemiology in Poland. The absence of a seasonal increase, despite a shift toward RSV-B predominance in 2024/25, suggests that subgroup dominance alone does not necessarily drive dual infection dynamics. Instead, sustained co-circulation of both RSV subgroups and ongoing population susceptibility may permit stable levels of coinfection across seasons. Although experimental and epidemiological studies indicate that infection with one RSV subgroup can induce transient heterologous immunity against the other, such protection may be incomplete, short-lived, or insufficient to entirely prevent mixed infections at the population level [46,47]. Further genetic and immunological investigations are warranted to determine whether coinfecting strains possess distinct antigenic or genomic characteristics that facilitate concurrent replication, and to clarify the clinical relevance of RSV-A/RSV-B coinfections, including their potential impact on disease severity and implications for naturally acquired or vaccine-induced immunity.

The detection of RSV coinfections with influenza and SARS-CoV-2, though infrequent, highlights the complexity of the post-pandemic respiratory virus landscape. The simultaneous circulation of multiple respiratory pathogens can affect disease burden and complicate clinical management, while viral interference may influence epidemic timing and intensity [48,49,50]. Integrating multiplex molecular surveillance will be crucial to understand these interactions and anticipate overlapping epidemic peaks that could challenge healthcare systems [51]. Higher RSV/influenza virus coinfection rates (4–7%) than RSV/SARS-CoV-2 ones (2–5%) likely reflect the epidemiological dynamics of these pathogens in the Polish population. According to national data and real-world studies of hospitalized patients, COVID-19 waves, which occurred in late summer or early autumn, usually precede the rise in influenza and RSV infections [52,53,54]. The latter typically coincide from December to January, peak in February, and decline by early April, making the coinfections with these pathogens more plausible.

Our findings provide epidemiological data relevant to immunization strategies. With maternal RSV vaccination and infant prophylaxis (nirsevimab) now available in many countries [55], understanding regional patterns of RSV subgroups is especially relevant. Although current vaccines and monoclonal antibodies target conserved F protein epitopes [56], ongoing subgroup shifts and potential antigenic evolution should be monitored to ensure continued efficacy. Notably, RSV-B infections were more prevalent among adults aged ≥ 60 years in the latter epidemic seasons, highlighting a potential age-specific subgroup pattern that may be particularly relevant for older populations targeted by emerging RSV vaccination and prevention strategies. Real-time molecular and genomic surveillance will be essential to detect any changes that could affect cross-protection or vaccine performance. The overlapping circulation of RSV, SARS-CoV-2, and influenza viruses further underscores the need for effective prophylaxis and supports efforts to develop combined vaccines to improve immunization protocols and logistics [57]. Moreover, based on our findings, we argue that studies evaluating real-world vaccine effectiveness should also include RSV subgroup-stratified analyses, coupled with viral subgroup surveillance throughout the epidemic season. Such an approach would allow for a better understanding of how shifts between RSV-A and RSV-B could influence prophylactic measures.

Several limitations of this study should be noted. First, RSV-positive samples were obtained through the routine VSES surveillance system, which provided broad geographic and temporal coverage but was not based on systematic sampling, so selection bias cannot be excluded. Most samples came from patients with respiratory symptoms undergoing routine diagnostic testing, which may not capture the full spectrum of RSV infections, including asymptomatic or mild cases. Second, the age distribution of cases varied considerably across epidemic seasons, with the mean age increasing from 7 years in 2022/23 to 33 years in 2024/25. This uneven age representation may reflect post-pandemic changes in RSV epidemiology, but could also be influenced by evolving testing practices, healthcare-seeking behavior, or surveillance characteristics that could not be assessed due to the lack of detailed clinical metadata. During and immediately after the COVID-19 pandemic, limited circulation of RSV and reduced exposure among pregnant women and young children have been proposed to contribute to transient immunity gaps, followed by increased pediatric case detection once social restrictions were lifted, as reported in Poland and other settings [13,14,58]. Consequently, the 2022/23 and 2023/24 samples included a disproportionately high number of children, which may influence observed subgroup distributions and limit direct comparability between seasons. Third, molecular characterization was limited to subgroup typing (RSV-A or RSV-B) using real-time qRT-PCR, without information on genotypic variability or evolutionary changes within subgroups. Additional sequencing would be needed to assess molecular diversity and phylogenetic relationships. Consequently, it was not possible to determine whether recurrent RSV-A or RSV-B detections represented newly emerged or previously circulating lineages, highlighting the need for routine whole-genome sequencing and international data sharing to support strain tracking and epidemic prediction. Fourth, no data on disease severity were available, preventing assessment of associations between RSV subgroup and clinical outcomes. Previous studies have reported inconsistent findings on infection severity, with some suggesting greater severity from RSV-A [44,45], others from RSV-B [59,60], and recent reviews indicating that genotype-specific differences may be more important than subgroup-level differences [8]. Most of these studies have focused on pediatric populations, so it remains unclear how age, immunosenescence, or comorbidities affect these relationships in adults [2]. Further research is needed, especially given our finding that RSV-B infections were more frequent among the elderly than among younger adults and children. Finally, as our study covered only three consecutive epidemic seasons (2022/23, 2023/24, and 2024/25), ongoing surveillance is required to determine long-term trends in RSV-A and RSV-B dominance and co-circulation.

## 5. Conclusions

This study provides the first molecular insights into the post-pandemic circulation of RSV subgroups in Poland, revealing dynamic shifts, increasing coinfection rates, and alignment with global epidemiological variability, though contrasting with observations from particular seasons in other regions of the world. These findings emphasize that trends reported elsewhere cannot necessarily be extrapolated to regional settings. Continued, integrated surveillance combining in-depth genomic, clinical, and epidemiological data will be essential to understand the drivers of subgroup alternation, its impact on disease severity, and its implications for vaccine policy, epidemic preparedness, and public health planning.

## Figures and Tables

**Table 1:** Basic demographic characteristics of the studied RSV-infected individuals.

	Season 2022/23 (*n* = 130)	Season 2023/24 (*n* = 112)	Season 2024/25 (*n* = 135)	*p*-Value
**Age**, mean ± SD (min–max), years	7.4 ± 17.2 (0–82) ^a^	16.9 ± 25.4 (0–82) ^b^	32.6 ± 31.2 (0–92) ^c^	<0.0001
**Age groups**				0.001
0–5, %	86.9	63.4	39.5	
6–17, %	3.1	12.5	8.5	
18–59, %	6.9	13.4	23.3	
≥60, %	3.1	10.7	28.7	
**Men/Women, %**	47.7/52.3	52.3/47.7	51.2/48.8	0.767

Different letters (a, b, c) in superscript denote statistically significant differences in age between groups, demonstrated with the post hoc Dunn’s test following the Kruskal–Wallis ANOVA (*p* < 0.05).

## Data Availability

The data that support the findings of this study are available from the corresponding author upon reasonable request.
